# Intra-Aortic Balloon Pump Use in Post-Infarction Ventricular Septal Rupture: The Impact of Timing Relative to Cardiogenic Shock

**DOI:** 10.3390/jcm15082892

**Published:** 2026-04-10

**Authors:** Si Wang, Qianfeng Xiao, Fangyang Huang, Yuan Feng, Jun Shi, Siyu He, Ying Xu, Xin Wei

**Affiliations:** 1 Department of Cardiology, West China Hospital, Sichuan University, 37 Guoxue Road, Chengdu 610041, China; si.wang@scu.edu.cn (S.W.);; 2 Department of Cardiac Surgery, West China Hospital, Sichuan University, 37 Guoxue Road, Chengdu 610041, China

**Keywords:** ventricular septal rupture, acute myocardial infarction, cardiogenic shock, intra-aortic balloon pump, timing

## Abstract

**Background**: Ventricular septal rupture (VSR) following acute myocardial infarction (AMI) creates an abrupt left-to-right shunt that can progress to cardiogenic shock (CS). Once CS develops, mortality increases dramatically and delayed repair becomes less feasible. Intra-aortic balloon pumps (IABPs) are widely used to facilitate delayed repair; however, whether initiating IABP before CS onset improves survival remains unclear. **Methods**: We retrospectively analyzed 124 patients with AMI-related VSR (2009–2024), categorized by IABP timing relative to CS onset (defined as first catecholamine administration) into pre-CS, post-CS, and no-IABP groups. The primary outcome was all-cause mortality within 90 days after AMI onset. Kaplan–Meier curves and Cox proportional hazards models were applied, with subgroup analyses by CS status. **Results:** The 90-day survival rate was 68.2% in the pre-CS IABP group, 14.3% in the post-CS group, and 35.1% in the no-IABP group. Pre-CS IABP was associated with significantly lower mortality compared with no-IABP (adjusted HR = 0.401, 95% CI 0.174–0.925, *p* = 0.032) and post-CS IABP (adjusted HR = 0.369, 95% CI 0.149–0.910, *p* = 0.030). In the CS subgroup, IABP use did not improve survival (19.4% vs. 17.6%, *p* = 0.365). Among non-CS patients, IABP use was independently associated with lower mortality (85.7% vs. 50.0%, *p* = 0.027; adjusted HR = 0.178, 95% CI 0.040–0.801, *p* = 0.025). **Conclusions**: Given the retrospective design and limited sample size, these findings are hypothesis-generating. Early IABP use was associated with improved short-term survival, an effect not observed once CS had developed. These findings support early risk stratification to identify high-risk patients who may benefit from timely hemodynamic support.

## 1. Introduction

Ventricular septal rupture (VSR) is a rare but catastrophic mechanical complication of acute myocardial infarction (AMI). Identified risk factors include advanced age, female sex, delayed revascularization, and the absence of timely reperfusion therapy [1]. Although the incidence of VSR has declined from approximately 2% to less than 0.3% due to widespread use of early reperfusion strategies [2,3,4], in-hospital mortality remains exceedingly high. Conservative management is associated with dismal outcomes, with survival rates reported to be below 10% [2,4], underscoring the role of surgical repair as the cornerstone of treatment. However, emergency surgery—while often necessary in unstable patients—carries high operative mortality. If hemodynamic status allows, delayed repair following a period of myocardial scarring may improve procedural success and outcomes [5,6]. VSR creates an abrupt left-to-right shunt, resulting in right ventricular volume overload, reduced left ventricular forward output, and progressive hemodynamic deterioration that may culminate in cardiogenic shock (CS) [4]. CS occurs in over half of AMI-related VSR cases and is a major determinant of early mortality [3,7,8]. However, our previous work demonstrated that not all patients develop CS immediately; a proportion remain hemodynamically stable at VSR diagnosis but subsequently deteriorate [9], suggesting a pre-shock window during which timely intervention could attenuate the progression to CS. Once CS develops, the feasibility of delayed repair is substantially reduced, and emergent intervention is often required. In these cases, intra-aortic balloon pump (IABP) support is frequently employed to stabilize hemodynamics, prolong the preoperative window, and facilitate definitive treatment. In more severe cases, advanced mechanical circulatory support (MCS) devices such as veno-arterial extracorporeal membrane oxygenation (VA-ECMO), Impella, or TandemHeart may be considered [4,10,11,12]. Despite mixed evidence in AMI-related CS, IABPs remain widely used in VSR due to their safety, simplicity, and cost-effectiveness [4,10,11,12].

Despite their widespread use, the optimal timing for IABP implantation and the specific populations most likely to benefit remain undefined. While prior registry studies have reported IABP utilization and outcomes in AMI-related VSR, none have stratified patients by the timing of IABP initiation relative to CS onset [8,13,14,15,16]. Given the rarity of this condition and the challenges of conducting large randomized controlled trials, we conducted a retrospective cohort study at a tertiary referral center to evaluate whether the timing of IABP initiation was associated with short-term survival in patients with AMI-related VSR. We also assessed the effect of IABP use in patients stratified by the in-hospital occurrence of CS. We hypothesized that IABP implantation before the onset of CS would be associated with improved short-term survival compared with later or no implantation.

## 2. Materials and Methods

### 2.1. Study Design and Population

This was a retrospective, single-center cohort study conducted at West China Hospital, Sichuan University. Patients diagnosed with both AMI and VSR between September 2009 and August 2024 were screened for eligibility. The inclusion criteria included a confirmed discharge diagnosis of both AMI and VSR, with AMI retrospectively validated against the Universal Definition of Myocardial Infarction applicable at the time of presentation, and VSR confirmed by echocardiographic evidence of a septal defect with left-to-right shunt. Patients were excluded if VSR was attributed to infective endocarditis, congenital heart disease, or trauma, or if hospital admission occurred more than 30 days after AMI onset. The study was approved by the Biomedical Ethics Committee of West China Hospital, Sichuan University (Approval No. 2021–1770).

### 2.2. Data Collection

Demographic and clinical data were independently extracted from institutional electronic medical records by two investigators using a standardized case report form, with discrepancies resolved by a senior investigator; assessors were not blinded to outcomes given the retrospective design. The extracted variables included age, sex, height, weight, smoking history, and vital signs on admission (systolic blood pressure [SBP], diastolic blood pressure [DBP], and heart rate). Comorbidities such as hypertension, diabetes mellitus, coronary artery disease, and pneumonia were recorded from discharge summaries. Time intervals from AMI onset to hospital admission and from AMI onset to VSR diagnosis were documented in days. Infarct location was classified as anterior or inferior based on electrocardiographic findings. Whether VSR was diagnosed before admission was also recorded.

### 2.3. Diagnostic Evaluation and Interventions

Transthoracic echocardiography was reviewed to assess VSR location (anterior vs. inferior), defect size, and LV dimensions. The presence of moderate-to-severe mitral or tricuspid regurgitation was recorded. Coronary angiography was used to assess coronary artery stenosis, with significant lesions defined as ≥70% luminal narrowing. Primary percutaneous coronary intervention (PCI) was defined as revascularization performed within 12 h of symptom onset. Preoperative PCI was defined as any PCI performed prior to VSR repair, regardless of the interval from AMI onset. Data on the VSR repair strategy were collected, including surgical versus percutaneous approach. Repairs were classified as emergency if performed due to hemodynamic instability refractory to medical therapy and mechanical circulatory support, as documented in physician or operative notes. All other repairs were considered elective. The interval from AMI onset to VSR repair was recorded in days. IABP use was recorded, including timing of insertion (based on physician order time) and duration of support. Use of other MCS devices, such as VA-ECMO, was also documented. Patients were classified into three groups according to the temporal relationship between IABP insertion and CS onset, determined retrospectively from documented timestamps in the electronic medical record system: pre-CS IABP group, post-CS IABP group, and no IABP group.

### 2.4. Laboratory Assessments

Baseline laboratory data obtained at admission included creatine kinase-MB (CK-MB), troponin-T, N-terminal pro-B-type natriuretic peptide (NT-proBNP), lactate, creatinine, uric acid, glucose, alanine aminotransferase (ALT), aspartate aminotransferase (AST), triglycerides, total cholesterol, high-density lipoprotein cholesterol (HDL-C), and low-density lipoprotein cholesterol (LDL-C). All laboratory tests were performed at the hospital’s central laboratory using standardized methods.

### 2.5. Definitions and Outcome

AMI onset was defined as the first episode of chest pain lasting ≥30 min before admission, adjudicated by two independent physicians based on clinical documentation, biomarker profiles, and referral records when available. VSR timing was based on the earliest documentation of a new systolic murmur or echocardiographic confirmation. For referred patients, external echocardiographic records were used. CS was defined as sustained hypotension (SBP < 90 mmHg or need for vasopressors), pulmonary congestion, and signs of end-organ hypoperfusion, corresponding to stage C or above in the SCAI shock classification [17]. CS onset was defined as the time of first administration of catecholamines (epinephrine, norepinephrine, or dopamine). Immediate CS was defined as CS present at the time of VSR diagnosis. The primary outcome was all-cause mortality within 90 days after the onset of AMI, determined through structured telephone follow-up. Survival time for analysis was measured from AMI onset to the date of death or censoring at 90 days post-AMI onset, a time horizon selected to accommodate delayed repair strategies and capture the full spectrum of early mortality across both surgical and conservatively managed patients.

### 2.6. Statistical Analysis

Continuous variables were assessed for normality using the Shapiro–Wilk test. Variables meeting the normality assumption are expressed as mean ± standard deviation (SD) and were compared using one-way analysis of variance (ANOVA), followed by pairwise comparisons with the independent *t*-test. Non-normally distributed variables are expressed as the median (interquartile range [IQR]) and were compared using the Kruskal–Wallis test, followed by pairwise comparisons with the Mann–Whitney U test. Bonferroni correction was applied for all pairwise comparisons, with an adjusted significance level of α = 0.0167. Categorical variables are presented as frequencies and percentages and were compared using Pearson’s chi-square test or Fisher’s exact test, as appropriate. Missing data were handled using complete case analysis. All covariates included in the Cox proportional hazards models had no missing data. The association between the timing of IABP implantation and all-cause mortality within 90 days after the onset of AMI was assessed using Kaplan–Meier analysis with the log-rank test and Cox proportional hazards regression models, with patients censored at 90 days after AMI onset if no death occurred. In the initial Cox model, the no-IABP group served as the reference to estimate hazard ratios (HRs) and 95% confidence intervals (CIs) for pre-CS and post-CS implantation. Subsequently, the post-CS IABP group served as the reference to assess the relative effect of pre-CS implantation. Multivariable Cox models were adjusted for age, sex, timing of VSR diagnosis, and in-hospital CS status, selected a priori based on clinical relevance and to maintain model stability given the sample size. Laboratory markers were not included, as these reflect acute hemodynamic severity closely related to CS status already adjusted for in the model, and the limited sample size necessitated a parsimonious model. The proportional hazards assumption was evaluated using Schoenfeld residual tests for all Cox models. Model stability was assessed by calculating the events-per-variable (EPV) ratio. A sensitivity analysis was performed excluding patients who received additional VA-ECMO support. Subgroup analyses were performed according to in-hospital CS status (CS vs. non-CS), applying the same Kaplan–Meier and Cox regression, with the no-IABP group serving as the reference and models adjusted for age, sex, and timing of VSR diagnosis, within each subgroup. All analyses were conducted using R software (version 4.5.2; R Foundation for Statistical Computing, Vienna, Austria), with a two-sided *p* value < 0.05 considered statistically significant. Survival curves and bar plots were generated with GraphPad Prism (version 10.0; GraphPad Software, San Diego, CA, USA).

## 3. Results

### 3.1. Baseline Characteristics According to Timing of IABP Implantation

**Figure 1:**
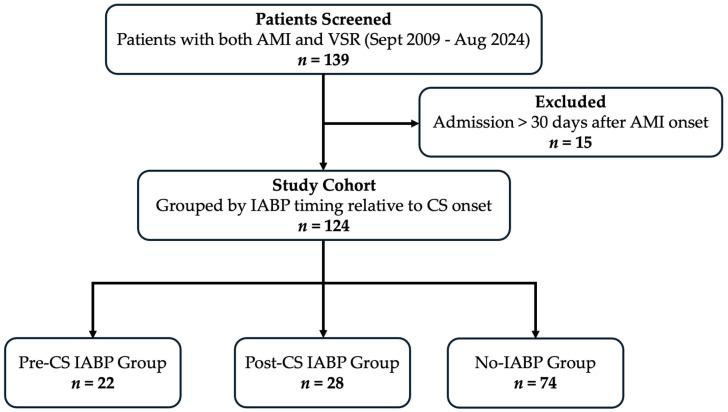
Patient flow diagram. AMI, acute myocardial infarction; VSR, ventricular septal rupture; CS, cardiogenic shock; IABP, intra-aortic balloon pump.

Of the 139 patients diagnosed with VSR after AMI, 124 met the inclusion criteria (Figure 1), with a median age of 70.0 years (IQR 62.0–77.0) and 54.8% of patients being male. Overall, 40 patients (32.3%) underwent VSR repair, and 45 (36.3%) survived beyond 90 days after AMI onset. Based on IABP timing, 22 patients (17.7%) received IABP support before CS onset (pre-CS group) and 28 (22.6%) after CS onset (post-CS group), and 74 (59.7%) did not receive IABP support. Only one patient in the post-CS IABP group received additional VA-ECMO support.

Baseline demographics, vital signs, and infarct and VSR characteristics were generally comparable across the three groups (Table 1). However, notable differences were observed in smoking status, prevalence of hypertension, laboratory markers of acute disease severity, Killip class, PCI history, and length of hospital stay. Pairwise comparisons indicated that the post-CS group exhibited the most disrupted laboratory profiles, with significantly higher CK-MB, troponin-T, and lactate compared with the pre-CS group. The pre-CS group had the longest AMI-to-admission intervals and the highest proportion of VSR diagnosed before admission, which may reflect referral bias, as these patients survived interhospital transfer and likely presented with more stable hemodynamics. Detailed pairwise comparisons are presented in Table 1.

### 3.2. Primary Outcomes and Survival Analysis by IABP Timing

In the overall cohort, the pre-CS IABP group demonstrated the highest 90-day post-AMI survival (68.2%), significantly exceeding the post-CS (14.3%) and no-IABP (35.1%) groups. This survival advantage was paralleled by the highest VSR repair rate in the pre-CS group (63.6%) compared with the post-CS (25.0%) and no-IABP (25.7%) groups. Notably, in the pre-CS group (*n* = 22), eight patients (36.4%) progressed to CS despite early IABP support; of these, three underwent delayed repair and five died.

**Figure 2:**
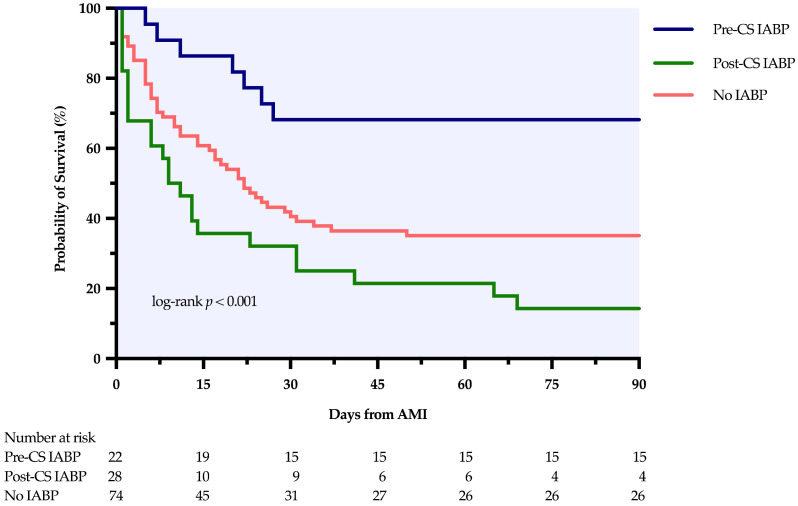
Kaplan–Meier survival analysis for 90-day mortality stratified by IABP timing. CS, cardiogenic shock; IABP, intra-aortic balloon pump; AMI, acute myocardial infarction.

Kaplan–Meier analysis showed significant differences in 90-day post-AMI survival among the three groups (log-rank *p* < 0.001; Figure 2). In Cox regression using the no-IABP group as the reference, the pre-CS IABP group had a significantly lower mortality risk (unadjusted HR = 0.358, 95% CI 0.162–0.791, *p* = 0.011; adjusted HR = 0.401, 95% CI 0.174–0.925, *p* = 0.032), whereas the post-CS IABP group did not show a significant survival benefit (unadjusted HR = 1.720, 95% CI 1.052–2.810, *p* = 0.031; adjusted HR = 1.089, 95% CI 0.628–1.888, *p* = 0.763). Using the post-CS IABP group as the reference, pre-CS implantation was also associated with a markedly lower mortality risk compared with post-CS implantation (unadjusted HR = 0.208, 95% CI 0.089–0.484, *p* < 0.001; adjusted HR = 0.369, 95% CI 0.149–0.910, *p* = 0.030). All multivariate models were adjusted for age, sex, timing of VSR diagnosis, and in-hospital CS status (Table 2).

The proportional hazards assumption was evaluated using Schoenfeld residuals. The global test was significant for the primary adjusted model (*p* = 0.004), primarily driven by time-varying effects of CS status (*p* = 0.022) and timing of VSR diagnosis (*p* = 0.009); the assumption was not violated for the primary exposure variable (IABP timing, *p* = 0.812). A sensitivity analysis using stratified Cox models (stratified by CS status and timing of VSR diagnosis) yielded consistent results: pre-CS IABP remained associated with lower mortality compared with no IABP (HR = 0.430, 95% CI 0.186–0.999, *p* = 0.050) and post-CS IABP (HR = 0.384, 95% CI 0.155–0.948, *p* = 0.038), with the proportional hazards assumption satisfied (global *p* = 0.152). Excluding the one patient who received VA-ECMO support also yielded consistent results (pre-CS vs. no IABP: adjusted HR = 0.402, *p* = 0.032; pre-CS vs. post-CS: adjusted HR = 0.372, *p* = 0.032). The EPV ratio for the primary model was 13.2 (79 events/6 parameters), indicating adequate model stability.

### 3.3. Subgroup Characteristics by CS Status and IABP Use

Of the 124 included patients, 70 (56.5%) developed CS and 54 (43.5%) did not. In the CS subgroup, 36 patients (51.4%) received IABP; in the non-CS subgroup, 14 (25.9%) received IABP. Baseline demographics, vital signs, and most comorbidities were largely similar between IABP and no-IABP patients within each CS stratum (Table 3). In the CS subgroup, notable differences included higher troponin-T and creatinine levels, more frequent primary and preoperative PCI, and a higher proportion of VSR diagnosed before admission in IABP-treated patients. In the non-CS subgroup, IABP-treated patients were more frequently female, shorter in stature, had higher rates of diabetes, and had lower total cholesterol and LDL-C; they also underwent more surgical repairs and had longer hospital stays. Detailed comparisons are presented in Table 3.

### 3.4. Survival Analysis by CS Status and IABP Use

**Figure 3:**
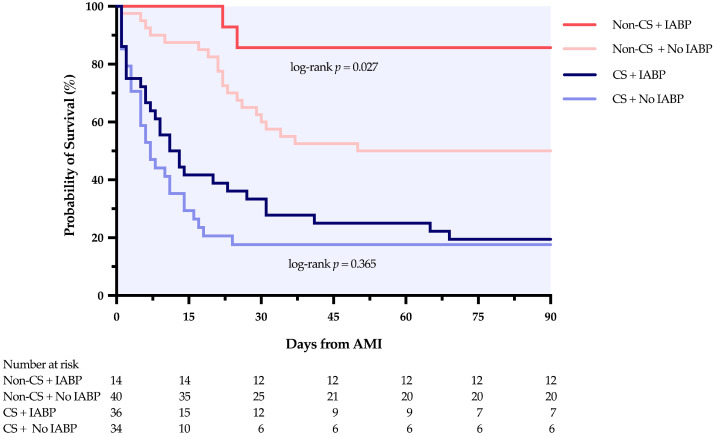
Kaplan–Meier survival analysis for 90-day mortality by CS status and IABP use. CS, cardiogenic shock; IABP, intra-aortic balloon pump; AMI, acute myocardial infarction.

In the CS subgroup, 90-day post-AMI survival did not differ significantly between the IABP and no-IABP groups (19.4% vs. 17.6%; log-rank *p* = 0.365) (Figure 3). Similarly, VSR repair rates were comparable between the two groups (30.6% vs. 11.8%; *p* = 0.081). Of the 36 IABP-treated CS patients, 7 survived beyond 90 days. Among the 29 deaths, 7 resulted from acute complications or failed repair, and 22 occurred after families declined escalation to advanced MCS or emergency surgery.

In the non-CS subgroup, the 90-day post-AMI survival was significantly higher in the IABP group compared with the no-IABP group (85.7% vs. 50.0%; log-rank *p* = 0.027) (Figure 3). This survival advantage was paralleled by a higher VSR repair rate in the IABP group (71.4%) compared with the no-IABP group (37.5%). Among the 14 IABP-treated patients, 2 declined repair and died after discharge. In the non-IABP group, 20 patients survived (14 repair, 6 conservative) and 20 died, of whom 17 declined repair and died after discharge. These patients were hemodynamically stable at discharge, and the potential impact of prolonged hospitalization remains uncertain.

Cox regression analysis showed that in the CS subgroup, IABP implantation was not associated with a statistically significant reduction in mortality risk in either univariate (HR = 0.787, 95% CI 0.467–1.326, *p* = 0.368) or multivariate models (HR = 1.069, 95% CI 0.613–1.863, *p* = 0.814). In contrast, in the non-CS subgroup, IABP implantation was associated with a significantly lower mortality risk in both univariate (HR = 0.226, 95% CI 0.053–0.967, *p* = 0.045) and multivariate analyses (HR = 0.178, 95% CI 0.040–0.801, *p* = 0.025). Covariates in the multivariate models included age, sex, and timing of VSR diagnosis (Table 4). The proportional hazards assumption was satisfied in both subgroups (CS: global *p* = 0.39; non-CS: global *p* = 0.23). The EPV ratio was 14.2 for the CS subgroup (57 events/4 parameters) and 5.5 for the non-CS subgroup (22 events/4 parameters); findings from the latter should therefore be considered exploratory, and the risk of type I error cannot be excluded.

## 4. Discussion

Despite substantial advances in MCS technologies that have improved the feasibility of delayed repair in patients with VSR following AMI, overall mortality remains 30% to 40% even after surgical intervention [1,13] and is even higher in those whose condition is complicated by CS [14,18,19]. Our previous work demonstrated that CS after VSR often evolves gradually: approximately two-thirds of patients are hemodynamically stable at diagnosis, yet nearly half of these subsequently develop delayed-onset CS [9]. This pattern highlights a potential therapeutic window during which timely intervention may potentially prevent hemodynamic collapse. However, current guidelines offer no specific recommendations on MCS use in VSR patients without CS. Although some experts advocate for early IABP implantation after VSR [12], its clinical benefit remains uncertain.

In this single-center retrospective study, we found that IABP implantation before CS onset was associated with significantly lower 90-day post-AMI mortality compared with both no IABP use and post-CS implantation. Although 36.4% of pre-CS IABP patients ultimately progressed to CS, their overall mortality remained lower than that of the other groups and lower than previously reported rates from other centers [1,13,14,15,16,18,19], suggesting that early hemodynamic support may stabilize patients and delay or prevent deterioration. These findings refine prior registry observations by identifying a clinically relevant time window in which proactive MCS initiation may improve prognosis.

### 4.1. Hemodynamic Rationale for Early IABP Use

The hemodynamic consequences of VSR are profound, characterized by an abrupt left-to-right shunt that causes an acute rise in ventricular pressures and volume load, leading to reduced cardiac output, hypotension, oliguria, and progression to CS [4]. Sympathetic activation compounds these effects by further increasing afterload and LV wall stress, thereby worsening the shunt and precipitating pulmonary congestion and right ventricular (RV) failure—both established predictors of adverse outcomes [4]. In this setting, IABP support can reduce LV afterload, enhance coronary perfusion, and lower myocardial wall stress [20]. For patients with AMI-related VSR, although IABP itself is not a definitive curative therapy, early IABP initiation—before the onset of CS—may lessen shunt magnitude, improve cardiac output, and reduce the risk of severe pulmonary edema and RV failure, thereby potentially preventing CS and creating a more favorable window for delayed repair [12]. This observation is consistent with the finding that the survival advantage in the pre-CS group was likely mediated, at least in part, by the significantly higher rate of VSR repair (63.6%). Early hemodynamic stabilization through IABP may have enabled more patients to reach delayed surgical repair, which in turn improved outcomes. This observation suggests that IABPs may function not only as direct hemodynamic support but also as a bridge enabling more patients to survive to definitive repair. Although the IABP-SHOCK II trial demonstrated no survival benefit in AMI with established CS [21], it underscored the unresolved question of optimal implantation timing [22,23], which may be particularly critical in AMI with mechanical complications such as VSR. Similar controversies also surround the initiation of VA-ECMO [24,25].

### 4.2. Limited Efficacy of IABP in Established CS

In the overall cohort, IABP implantation after CS onset was likewise not associated with a statistically significant reduction in 90-day post-AMI mortality compared with no IABP use. Similarly, in the subgroup of patients who developed CS during hospitalization, IABP support did not significantly improve outcomes regardless of implantation timing (pre- or post-CS), with most deaths resulting from persistent hemodynamic collapse. Although the 2023 ESC guidelines recommend that IABP use should be considered in patients with CS or hemodynamic instability due to mechanical complications (Class IIa, Level of Evidence C) [10], our findings suggest that once CS has developed, IABP support alone may be insufficient to rescue critically ill CS patients, as the observed numerical survival advantage did not reach statistical significance. This observation is broadly consistent with the IABP-SHOCK II trial [21] and with the 2025 ACC/AHA guideline recommendation against the routine use of IABPs in patients with AMI and CS (Class 3: No Benefit, Level of Evidence B-R) [26].

The 2025 ACC/AHA guideline additionally endorses the use of short-term MCS devices for hemodynamic stabilization as a bridge to surgery in patients with mechanical complications of AMI (Class 2a, Level of Evidence B-NR) [26]; however, the optimal device selection remains unspecified. VA-ECMO can provide biventricular support and improve systemic perfusion, but may increase LV afterload and pulmonary pressures, potentially delaying myocardial recovery and exacerbating the shunt [12,27]. Impella offers superior hemodynamic support, reduces shunting, and alleviates RV overload; however, it carries risks including suction-related necrosis, septal injury, and systemic hypoxemia due to shunt reversal [4]. TandemHeart unloads the LV via left atrial drainage and avoids suction-related complications but may lead to excessive unloading, shunt reversal, and cerebral hypoxia [28]. Overall, although these advanced MCS modalities can stabilize systemic perfusion and blood pressure, none can fully correct the hemodynamic derangements of VSR, and the optimal device selection remains uncertain due to limited comparative data [12,19,26]. Given its safety profile, simplicity, and cost-effectiveness, IABPs remain widely used in clinical practice. In our center, shared decision-making with elderly patients and their families often favored less invasive approaches over aggressive MCS escalation or emergency surgery, which likely contributes to the persistently poor outcomes observed in this subgroup. The high rate of hemodynamic deterioration despite ongoing IABP support in these patients further underscores the limited efficacy of IABP support alone once CS is established, and highlights the need for timely escalation to more advanced support or emergency repair.

### 4.3. Benefit of IABP Support in Hemodynamically Stable Patients

Beyond the overall survival benefit of pre-CS IABP, subgroup analysis demonstrated that this advantage was most evident in patients who remained free of CS during hospitalization, in whom IABP use was independently associated with better outcomes. These findings suggest that in selected hemodynamically stable patients, early mechanical unloading and improved coronary perfusion may interrupt the progression toward circulatory collapse, thereby enabling more patients to survive to undergo delayed repair, as reflected by the higher VSR repair rate among IABP-treated non-CS patients (71.4% vs. 37.5%). In contrast, this beneficial association appears to be lost once CS is established, likely compounded by a higher rate of treatment abandonment among these critically ill patients. Of note, the 2025 ACC/AHA guideline recommendation for short-term MCS in mechanical complications of AMI is not contingent on the presence of CS [26]; yet to our knowledge, no previous studies have specifically examined IABP use in VSR patients without CS. Registry data from other countries show that IABP utilization often exceeds the incidence of CS [13,14,29], suggesting that some hemodynamically stable patients receive preventive support. Conversely, Chinese studies report lower IABP usage rates [8,15,16], likely reflecting resource limitations and differences in clinical decision-making.

**Figure 4:**
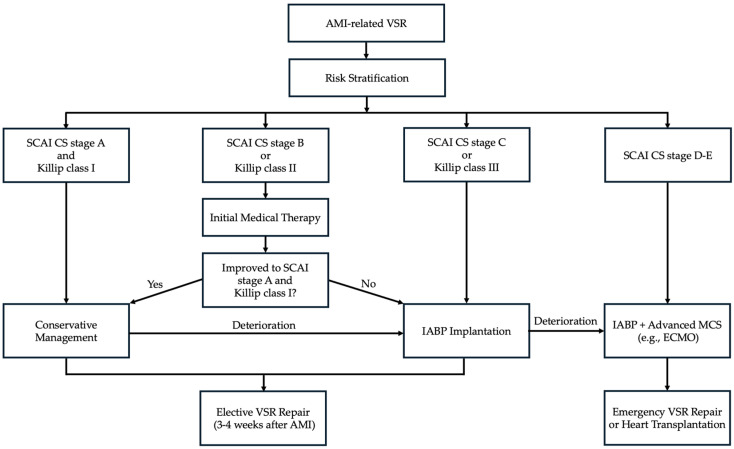
Institutional algorithm for IABP use in patients with AMI-related VSR, stratified by SCAI shock classification and Killip class. AMI, acute myocardial infarction; VSR, ventricular septal rupture; SCAI, Society for Cardiovascular Angiography and Interventions; IABP, intra-aortic balloon pump; MCS, mechanical circulatory support; ECMO, extracorporeal membrane oxygenation.

Importantly, however, our data also show that half of the patients who did not develop CS survived without IABPs, suggesting that routine implantation may not be necessary for all VSR patients [12]. This finding, together with our previous observation that not all patients develop CS immediately after AMI-related VSR—some experience delayed onset while others remain free of CS throughout hospitalization [9]—indicates that early IABP may help attenuate hemodynamic deterioration in selected high-risk patients, whereas lower-risk patients can be managed conservatively until elective repair. Accurate risk stratification is therefore essential; however, existing risk models have not been prospectively validated [30,31], and no standardized algorithm currently exists for early MCS use in this population [10]. Based on the above evidence, our center employs a selective IABP strategy stratified by SCAI shock classification and Killip class, with stepwise escalation from conservative management to IABP support and advanced MCS as hemodynamic status dictates. This institutional algorithm is summarized in Figure 4.

### 4.4. Limitations

This retrospective single-center study is subject to inherent selection bias and confounding by indication, as treatment decisions regarding IABP use were driven by physician judgment and family preferences. Patients who received pre-CS IABP may have been selected because they were perceived as high-risk but still hemodynamically stable enough to tolerate the procedure, representing a prognostically distinct subgroup. Although key covariates were adjusted for in multivariable models, residual confounding from unmeasured factors—such as VSR size, laboratory markers of disease severity, and family treatment preferences—may persist. A proportion of patients were transferred from other hospitals, and those in the pre-CS group had longer AMI-to-admission intervals and a higher proportion of VSR diagnosed before admission. This pattern, together with immortal time bias—whereby patients must survive without CS long enough to receive pre-CS IABP—may confer an inherent survival advantage unrelated to the intervention itself. CS was defined as SCAI stage C or above, with onset operationalized as the time of first catecholamine administration. This definition may not capture all cases of early hemodynamic compromise, and misclassification of IABP timing relative to CS onset cannot be excluded. Temporal changes in clinical practice over the 15-year study period may also have influenced outcomes, although calendar year was not included as a covariate to preserve model stability. Finally, the relatively small number of patients receiving IABP support—especially when stratified by timing—yielded wide confidence intervals, warranting cautious interpretation of effect size estimates. In particular, the non-CS subgroup model had an EPV ratio of 5.5, and these results should be considered exploratory.

## 5. Conclusions

Given the limitations of this retrospective study, our findings are hypothesis-generating. They suggest that IABP implantation before the onset of CS may stabilize hemodynamics, delay deterioration, and improve short-term outcomes in AMI-related VSR, an effect not observed once CS is established. These findings should not be interpreted as evidence for routine prophylactic IABP use in all hemodynamically stable VSR patients, but rather support early risk stratification to identify high-risk patients who may benefit from timely hemodynamic support. VSR leads to acute biventricular dysfunction, which may progress to cardiogenic shock and chronic heart failure; the long-term impact of early mechanical circulatory support on ventricular remodeling and heart failure progression remains unexplored [32]. Future research should focus on validating risk stratification tools, optimizing the timing of MCS initiation, and comparing different MCS modalities in this population. Prospective multicenter registries and pragmatic clinical trials are warranted to address these questions.

## Figures and Tables

**Table 1:** Baseline characteristics according to timing of IABP implantation.

	Pre-CS IABP (*n* = 22)	Post-CS IABP (*n* = 28)	No IABP (*n* = 74)	Total (*n* = 124)	*p* Value
Age, years	69.0 (62.1–72.8)	73.0 (64.5–78.2)	71.5 (61.2–77.8)	70.0 (62.0–77.0)	0.315
Male	13 (59.1%)	13 (46.4%)	42 (56.8%)	68 (54.8%)	0.586
Height, cm	161.1 ± 6.6	158.6 ± 7.9	162.4 ± 7.9 ^b^	161.3 ± 7.8	0.094
Weight, kg	65.0 (58.0–74.0)	60.0 (55.0–67.2)	62.2 (55.0–69.0)	62.0 (55.0–69.0)	0.446
BMI, kg/m^2^	25.0 (21.9–26.9)	23.8 (21.2–25.9)	23.4 (21.5–25.4)	24.0 (21.4–26.3)	0.369
SBP, mmHg	107.0 (104.0–113.8)	98.0 (87.8–114.0)	106.0 (95.0–116.0)	105.5 (94.0–115.2)	0.172
DBP, mmHg	73.0 (68.0–77.8)	65.5 (56.2–76.8)	70.0 (61.0–80.0)	70.0 (60.0–79.2)	0.131
Heart rate, beats/min	104 ± 19.4	102.1 ± 18.3	102.3 ± 18.0	102.5 ± 18.2	0.918
Smoking history	10 (45.5%)	4 (14.3%) ^a^	34 (45.9%) ^b^	48 (38.7%)	0.008
Hypertension	16 (72.7%)	12 (42.9%) ^a^	31 (41.9%) ^a^	59 (47.6%)	0.034
Diabetes mellitus	12 (54.5%)	8 (28.6%)	22 (29.7%) ^a^	42 (33.9%)	0.077
Coronary artery disease	3 (13.6%)	2 (7.1%)	4 (5.4%)	9 (7.3%)	0.390
Pneumonia	17 (77.3%)	18 (64.3%)	45 (60.8%)	80 (64.5%)	0.366
Anterior infarction	14 (63.6%)	19 (67.9%)	58 (78.4%)	91 (73.4%)	0.293
VSR location					
Anterior	5 (23.8%)	5 (17.9%)	14 (20.0%)	24 (20.2%)	0.542
Posterior	10 (47.6%)	9 (32.1%)	22 (31.4%)	41 (34.5%)	
Apical	6 (28.6%)	14 (50.0%)	34 (48.6%)	54 (45.4%)	
Size of VSR, mm	17.5 (11.0–21.8)	17.0 (13.6–20.0)	13.5 (10.0–18.0)	15.0 (11.0–20.0)	0.057
LVEDD, mm	49.5 (47.0–54.0)	48.5 (43.5–51.5)	51.0 (47.0–54.0)	50.0 (47.0–53.0)	0.093
LVEF, %	45.6 ± 12.7	50.2 ± 12.3	47.9 ± 12.1	48 ± 12.3	0.430
Mitral regurgitation	7 (31.8%)	7 (25.0%)	21 (29.2%)	35 (28.7%)	0.861
Tricuspid regurgitation	15 (68.2%)	17 (60.7%)	40 (55.6%)	72 (59.0%)	0.562
CK-MB, ng/mL	4.1 (2.5–27.5)	56.6 (9.3–195.9) ^a^	6.9 (3.0–23.8) ^b^	8.1 (3.4–56.6)	<0.001
Troponin-T, ng/mL	1777.0 (596.3–3368.0)	4604.0 (2401.5–9070.5) ^a^	1391.5 (451.3–4131.2) ^b^	1794.0 (803.9–5173.0)	<0.001
NT-proBNP, ng/L	8177.5 (6568.5–19,607.5)	13,223.0 (7888.8–29,630.0)	8425.5 (3440.0–15,632.5)	9368.0 (4628.0–19,958.8)	0.076
Lactate, mmol/L	1.6 (1.5–1.8)	5.5 (2.5–9.8) ^a^	1.9 (1.6–3.7) ^b^	2.0 (1.6–4.8)	<0.001
Creatinine, µmol/L	115.5 (90.2–150.2)	149.0 (109.0–249.0)	103.5 (75.5–152.2) ^b^	114.0 (83.0–166.0)	0.019
Uric acid, µmol/L	449.0 (359.0–608.5)	488.0 (315.2–577.0)	433.4 (335.0–565.5)	455.0 (335.5–576.0)	0.814
ALT, U/L	71.0 (43.5–401.0)	79.0 (40.5–544.0)	56.5 (26.8–97.0)	61.0 (32.0–152.0)	0.052
AST, U/L	182.0 (44.2–341.2)	399.0 (121.0–943.0)	70.5 (34.8–228.8) ^b^	130.0 (42.0–346.0)	<0.001
Glucose, mmol/L	9.3 (7.7–13.2)	12.0 (8.5–14.1)	9.5 (6.7–12.1)	9.7 (7.0–13.3)	0.082
Triglyceride, mmol/L	1.3 (1.1–1.5)	1.3 (0.9–1.5)	1.3 (1.0–1.7)	1.3 (0.9–1.6)	0.672
Total cholesterol, mmol/L	2.9 (2.4–3.5)	4.1 (3.2–4.7) ^a^	4.0 (3.5–4.6) ^a^	4.0 (3.1–4.5)	<0.001
HDL-C, mmol/L	0.9 (0.7–1.2)	1.1 (0.8–1.3)	1.0 (0.7–1.2)	1.0 (0.7–1.2)	0.414
LDL-C, mmol/L	1.6 (1.1–1.9)	2.6 (1.9–3.1) ^a^	2.4 (1.9–3.0) ^a^	2.3 (1.8–2.9)	<0.001
Killip class at admission					<0.001
I	0	0	4 (5.4%)	4 (3.2%)	
II	9 (40.9%)	2 (7.1%)	23 (31.1%)	34 (27.4%)	
III	13 (59.1%)	4 (14.3%)	31 (41.9%)	48 (38.7%)	
IV	0	22 (78.6%)	16 (21.6%)	38 (30.6%)	
AMI to admission time, days	12.0 (4.2–18.0)	1.7 (0.9–5.0) ^a^	5.0 (2.0–10.0) ^b^	5.0 (1.1–10.0)	<0.001
AMI to VSR time, days	4.8 (1.2–13.5)	2.0 (1.0–5.0)	5.0 (1.0–9.8)	4.0 (1.0–7.2)	0.058
VSR discovered before admission	13 (59.1%)	7 (25.0%) ^a^	22 (29.7%) ^a^	42 (33.9%)	0.020
Primary PCI	6 (27.3%)	8 (28.6%)	7 (9.5%) ^b^	21 (16.9%)	0.026
Preoperative PCI	14 (63.6%)	15 (53.6%)	19 (25.7%) ^a^	48 (38.7%)	<0.001
Angiographic data					
Negative	0	0	2 (5.0%)	2 (2.3%)	0.950
One-vessel disease	12 (54.5%)	14 (56.0%)	23 (57.5%)	49 (56.3%)	
Two-vessel disease	7 (31.8%)	7 (28.0%)	11 (27.5%)	25 (28.7%)	
Three-vessel disease	3 (13.6%)	4 (16.0%)	4 (10.0%)	11 (12.6%)	
Surgical VSR repair	13 (46.4%)	5 (17.9%) ^a^	10 (35.7%) ^a^	28 (22.6%)	<0.001
Percutaneous VSR repair	1 (4.5%)	2 (7.1%)	9 (12.2%)	12 (9.7%)	0.639
Emergency VSR repair	1 (4.5%)	2 (7.1%)	1 (1.4%)	4 (3.2%)	0.179
Time from AMI to VSR repair, days	25.0 (21.0–35.2)	22.0 (19.5–25.0)	35.0 (25.0–45.0)	27.0 (22.0–39.2)	0.049
Duration of IABP use, days	11.0 (9.0–16.5)	2.5 (1.0–9.2) ^a^	—	8.5 (1.2–13.0)	<0.001
CS	8 (36.4%)	28 (100%) ^a^	34 (45.9%) ^a^	70 (56.5%)	<0.001
Immediate CS	0	23 (82.1%) ^a^	16 (21.6%) ^b^	39 (31.5%)	<0.001
Length of hospital stay, days	21.0 (14.0–34.5)	3.5 (1.0–14.8) ^a^	6.0 (2.0–14.8) ^a^	8.0 (2.0–19.2)	<0.001

Variables with normal distribution confirmed by Shapiro–Wilk test are presented as mean ± SD and were tested by one-way ANOVA. All other variables are presented as median (IQR), tested by Kruskal–Wallis test. Abbreviations: CS, cardiogenic shock; IABP, intra-aortic balloon pump; Pre-CS, before CS onset; Post-CS, after CS onset; BMI, body mass index; SBP, systolic blood pressure; DBP, diastolic blood pressure; VSR, ventricular septal rupture; LVEDD, left ventricular end-diastolic diameter; LVEF, left ventricular ejection fraction; CK-MB, creatine kinase-MB; NT-proBNP, N-terminal pro-B-type natriuretic peptide; ALT, alanine transaminase; AST, aspartate transaminase; HDL-C, high-density lipoprotein cholesterol; LDL-C, low-density lipoprotein cholesterol; PCI, percutaneous coronary intervention. ^a^ *p* < 0.0167 vs. Pre-CS IABP group; ^b^ *p* < 0.0167 vs. Post-CS IABP group (Bonferroni-corrected pairwise comparisons using Mann–Whitney U test).

**Table 2:** Cox proportional hazards analysis for 90-day mortality in the overall cohort.

Variable	Unadjusted HR (95% CI)	*p* Value	Adjusted HR (95% CI) *	*p* Value
Reference = No IABP (*n* = 74)				
Pre-CS IABP (*n* = 22)	0.358 (0.162–0.791)	0.011	0.401 (0.174–0.925)	0.032
Post-CS IABP (*n* = 28)	1.720 (1.052–2.810)	0.031	1.089 (0.628–1.888)	0.763
Reference = Post-CS IABP (*n* = 28)				
Pre-CS IABP (*n* = 22)	0.208 (0.089–0.484)	<0.001	0.369 (0.149–0.910)	0.030
No IABP (*n* = 74)	0.582 (0.356–0.950)	0.031	0.919 (0.530–1.593)	0.763

Abbreviations: CS, cardiogenic shock; IABP, intra-aortic balloon pump; HR, hazard ratio; CI, confidence interval. * Adjusted for age, sex, timing of VSR diagnosis, and in-hospital CS status.

**Table 3:** Patient characteristics according to CS status and IABP use.

	Cardiogenic Shock (*n* = 70)				Non-Cardiogenic Shock (*n* = 54)			
	CS with IABP (*n* = 36)	CS Without IABP (*n* = 34)	Total	*p*1 Value	Non-CS with IABP (*n* = 14)	Non-CS Without IABP (*n* = 40)	Total	*p*2 Value
Age, years	71.0 (65.8–78.0)	76.0 (70.2–78.0)	74.0 (67.0–78.0)	0.116	66.3 (58.0–71.8)	66.0 (58.8–76.2)	66.0 (58.2–74.8)	0.745
Male	21 (58.3%)	11 (32.4%)	32 (45.7%)	0.029	5 (35.7%)	31 (77.5%)	36 (66.7%)	0.004
Height, cm	159.6 ± 7.5	159.6 ± 7.1	159.6 ± 7.3	0.993	159.9 ± 7.5	164.7 ± 7.8	163.4 ± 7.9	0.048
Weight, kg	62.0 (55.0–70.0)	61.0 (52.8–65.0)	61.0 (53.8–69.2)	0.602	63.0 (58.2–68.8)	65.0 (57.0–69.0)	64.0 (58.0–69.0)	0.748
BMI, kg/m^2^	24.0 (21.0–26.9)	23.8 (21.3–25.4)	23.9 (21.1–26.3)	0.705	24.8 (22.3–26.4)	23.4 (21.7–25.5)	24.0 (21.8–26.0)	0.318
SBP, mmHg	100.0 (90.2–110.8)	96.0 (88.8–111.5)	98.0 (88.8–111.5)	0.782	110.0 (106.2–115.5)	107.5 (103.5–117.8)	108.5 (104.2–116.8)	0.812
DBP, mmHg	66.5 (56.5–76.0)	66.0 (60.0–74.8)	66.5 (58.0–75.8)	0.823	74.5 (72.0–80.0)	73.0 (64.0–81.2)	73.5 (65.5–80.8)	0.671
Heart rate, beats/min	105.0 ± 19.1	109.5 ± 19.0	107.2 ± 19.0	0.332	97.6 ± 16.9	96.1 ± 14.8	96.5 ± 15.2	0.751
Smoking history	9 (25.0%)	11 (32.4%)	20 (28.6%)	0.496	5 (35.7%)	23 (57.5%)	28 (51.9%)	0.16
Hypertension	18 (50.0%)	12 (35.3%)	30 (42.9%)	0.214	10 (71.4%)	19 (47.5%)	29 (53.7%)	0.212
Diabetes mellitus	10 (27.8%)	12 (35.3%)	22 (31.4%)	0.498	10 (71.4%)	10 (25.0%)	20 (37.0%)	0.003
Coronary artery disease	2 (5.6%)	0 (0%)	2 (2.9%)	0.493	3 (21.4%)	4 (10.0%)	7 (13.0%)	0.358
Pneumonia	24 (66.7%)	19 (55.9%)	43 (61.4%)	0.354	11 (78.6%)	26 (65.0%)	37 (68.5%)	0.507
Anterior infarction	26 (72.2%)	28 (82.4%)	54 (77.1%)	0.313	7 (50.0%)	30 (75.0%)	37 (68.5%)	0.083
VSR location								
Anterior	7 (19.4%)	8 (24.2%)	15 (21.7%)	0.56	3 (23.1%)	6 (16.2%)	9 (18.0%)	0.641
Posterior	13 (36.1%)	8 (24.2%)	21 (30.4%)		6 (46.2%)	14 (37.8%)	20 (40.0%)	
Apical	16 (44.4%)	17 (51.5%)	33 (47.8%)		4 (30.8%)	17 (45.9%)	21 (42.0%)	
Size of VSR, mm	15.0 (10.2–19.0)	12.0 (9.5–15.5)	13.5 (10.0–17.0)	0.078	15.0 (11.0–17.8)	10.0 (8.0–17.0)	11.5 (8.8–17.0)	0.136
LVEDD, mm	49.0 (45.2–52.0)	48.5 (45.8–52.0)	49.0 (45.2–52.0)	0.892	49.5 (47.0–57.0)	52.0 (50.0–55.8)	52.0 (48.8–56.0)	0.358
LVEF, %	50.0 ± 13.5	47.3 ± 11.6	48.7 ± 12.6	0.375	43.4 ± 8.7	48.5 ± 12.7	47.1 ± 11.9	0.176
Mitral regurgitation	8 (22.2%)	6 (17.6%)	14 (20.0%)	0.632	9 (64.3%)	15 (39.5%)	24 (46.2%)	0.111
Tricuspid regurgitation	23 (63.9%)	23 (67.6%)	46 (65.7%)	0.741	6 (42.9%)	17 (44.7%)	23 (44.2%)	0.904
CK-MB, ng/mL	48.2 (8.2–191.8)	17.6 (6.9–106.5)	23.2 (7.1–163.9)	0.327	3.3 (2.0–5.0)	4.2 (1.9–7.6)	3.8 (1.9–7.4)	0.622
Troponin-T, ng/mL	4604.0 (2388.5–8182.5)	2144.0 (1131.0–5181.8)	3695.0 (1507.5–6650.0)	0.015	919.5 (269.9–2200.2)	828.8 (266.2–1938.2)	877.6 (260.6–1946.8)	0.664
NT-proBNP, ng/L	11,019.0 (7008.0–29,630.0)	10,344.5 (4914.0–21,277.8)	10,344.5 (6141.0–25,843.8)	0.429	9231.0 (7229.2–18,500.5)	6181.0 (2940.8–10,676.0)	7517.5 (3646.8–12,776.2)	0.046
Lactate, mmol/L	4.9 (1.6–7.8)	3.8 (1.8–6.5)	4.1 (1.7–7.6)	0.854	1.6 (1.5–1.9)	1.6 (1.4–2.1)	1.6 (1.5–2.0)	0.456
Creatinine, µmol/L	142.0 (109.0–249.0)	121.0 (72.0–174.8)	130.0 (89.5–199.0)	0.049	98.5 (81.2–144.0)	102.0 (77.8–124.0)	101.0 (77.2–126.0)	0.813
Uric acid, µmol/L	495.0 (326.5–600.5)	392.4 (338.8–556.5)	472.0 (336.2–593.0)	0.324	429.0 (359.0–471.0)	454.5 (328.2–565.5)	438.9 (336.2–564.8)	0.515
ALT, U/L	82.0 (47.0–678.0)	60.5 (28.0–88.0)	72.0 (38.5–248.5)	0.056	54.5 (38.2–165.5)	44.0 (21.8–115.0)	48.5 (26.2–117.0)	0.401
AST, U/L	327.0 (180.0–678.0)	114.0 (47.0–314.5)	237.0 (64.5–480.5)	0.011	93.5 (32.5–200.5)	45.5 (28.5–138.8)	50.5 (29.0–144.0)	0.374
Glucose, mmol/L	11.7 (8.5–14.0)	10.5 (8.1–15.6)	10.7 (8.3–14.1)	0.846	10.0 (7.7–13.0)	7.2 (6.3–10.3)	7.8 (6.4–11.6)	0.071
Triglyceride, mmol/L	1.2 (0.9–1.5)	1.2 (0.9–1.5)	1.2 (0.9–1.5)	0.856	1.3 (1.1–1.6)	1.5 (1.1–1.7)	1.4 (1.1–1.7)	0.459
Total cholesterol, mmol/L	4.0 (3.2–4.7)	4.0 (3.6–4.7)	4.0 (3.3–4.7)	0.449	2.8 (2.3–3.1)	4.0 (3.3–4.5)	3.6 (3.0–4.3)	<0.001
HDL-C, mmol/L	1.1 (0.8–1.3)	1.2 (0.9–1.3)	1.1 (0.8–1.3)	0.464	0.9 (0.7–1.1)	0.9 (0.7–1.1)	0.9 (0.7–1.1)	0.775
LDL-C, mmol/L	2.4 (1.9–3.1)	2.5 (2.1–2.9)	2.4 (1.9–3.0)	0.687	1.5 (1.1–1.7)	2.4 (1.9–3.0)	2.2 (1.6–2.8)	<0.001
Killip class at admission								0.361
I	0	0	0	0.337	0	4 (10.0%)	4 (7.4%)	
II	6 (16.7%)	5 (14.7%)	11 (15.7%)		9 (64.3%)	18 (45.0%)	27 (50.0%)	
III	8 (22.2%)	13 (38.2%)	21 (30.0%)		5 (35.7%)	18 (45.0%)	23 (42.6%)	
IV	22 (61.1%)	16 (47.1%)	38 (54.3%)		0	0	0	
AMI to admission time, days	2.5 (1.0–5.2)	2.0 (0.8–4.0)	2.0 (0.8–5.0)	0.498	14.5 (8.8–19.8)	10.0 (4.8–20.0)	10.0 (5.0–20.0)	0.181
AMI to VSR time, days	3.0 (1.0–5.0)	2.5 (1.0–6.0)	3.0 (1.0–6.0)	0.906	4.0 (1.0–13.5)	7.5 (2.0–13.2)	6.0 (2.0–13.8)	0.404
VSR discovered before admission	12 (33.3%)	4 (11.8%)	16 (22.9%)	0.046	8 (57.1%)	18 (45.0%)	26 (48.1%)	0.434
Primary PCI	11 (30.6%)	2 (5.9%)	13 (18.6%)	0.012	4 (28.6%)	3 (7.5%)	7 (13.0%)	0.065
Preoperative PCI	22 (61.1%)	6 (17.6)	28 (40.0%)	<0.001	7 (50.0%)	13 (32.5%)	20 (37.0%)	0.243
Angiographic data								
Negative	0 (%)	1 (8.3%)	1 (2.2%)	0.5	0 (%)	1 (3.6%)	1 (2.4%)	0.699
One-vessel disease	20 (60.6%)	7 (58.3%)	27 (60.0%)		6 (42.9%)	16 (57.1%)	22 (52.4%)	
Two-vessel disease	9 (27.3%)	3 (25.0%)	12 (26.7%)		5 (35.7%)	8 (28.6%)	13 (31.0%)	
Three-vessel disease	4 (12.1%)	1 (8.3%)	5 (11.1%)		3 (21.4%)	3 (10.7%)	6 (14.3%)	
Surgical VSR repair	8 (22.2%)	4 (11.8%)	12 (17.1%)	0.345	10 (71.4%)	6 (14.3%)	16 (28.6%)	<0.001
Percutaneous VSR repair	3 (27.3%)	0 (0%)	3 (20.0%)	0.516	0 (0%)	9 (60.0%)	9 (36.0%)	0.003
Emergency VSR repair	3 (8.3%)	0 (0%)	3 (4.5%)	0.245	0 (0%)	1 (2.5%)	1 (1.9%)	1
Time from AMI to VSR repair, days	23.0 (19.5–26.0)	36.5 (24.8–51.8)	25.0 (20.0–27.0)	0.131	25.5 (21.0–41.8)	35.0 (26.0–42.0)	34.0 (23.0–43.0)	0.488
Duration of IABP use, days	3.0 (1.0–12.2)	—	3.0 (1.0–12.2)	—	11.0 (9.0–16.5)	—	11.0 (9.0–16.5)	—
Immediate CS	23 (63.9%)	16 (47.1%)	39 (55.7%)	0.229	0	0	0	
Length of hospital stay, days	7.5 (1.0–19.0)	3.5 (1.0–8.2)	4.0 (1.0–13.8)	0.133	24.0 (20.2–35.8)	12.0 (5.0–17.0)	14.0 (6.2–21.0)	<0.001

Variables with normal distribution confirmed by Shapiro–Wilk test are presented as mean ± SD (tested by independent *t*-test). All other variables are presented as median (IQR) (Mann–Whitney U test). Abbreviations are as in Table 1.

**Table 4:** Cox proportional hazards analysis of IABP use for 90-day mortality stratified by CS status.

Variable	Unadjusted HR (95% CI)	*p* Value	Adjusted HR (95% CI) *	*p* Value
CS Subgroup (*n* = 70)				
No IABP (*n* = 34)	Reference		Reference	
IABP (*n* = 36)	0.787 (0.467–1.326)	0.368	1.069 (0.613–1.863)	0.814
Non-CS Subgroup (*n* = 54)				
No IABP (*n* = 40)	Reference		Reference	
IABP (*n* = 14)	0.226 (0.053–0.967)	0.045	0.178 (0.040–0.801)	0.025

Abbreviations: CS, cardiogenic shock; IABP, intra-aortic balloon pump; HR, hazard ratio; CI, confi dence interval. * Adjusted for age, sex, and timing of VSR diagnosis.

## Data Availability

The data presented in this study are not publicly available due to ethical and privacy restrictions. The data are available from the corresponding author upon reasonable request.

## Abbreviations

The following abbreviations are used in this manuscript:

ALT	Alanine aminotransferase
AMI	Acute myocardial infarction
AST	Aspartate aminotransferase
CI	Confidence interval
CK-MB	Creatine kinase-MB
CS	Cardiogenic shock
DBP	Diastolic blood pressure
HDL-C	High-density lipoprotein cholesterol
HR	Hazard ratio
IABP	Intra-aortic balloon pump
LDL-C	Low-density lipoprotein cholesterol
LV	Left ventricular
LVEDD	Left ventricular end-diastolic diameter
LVEF	Left ventricular ejection fraction
MCS	Mechanical circulatory support
NT-proBNP	N-terminal pro-B-type natriuretic peptide
PCI	Percutaneous coronary intervention
RV	Right ventricular
SBP	Systolic blood pressure
SCAI	Society for Cardiovascular Angiography and Interventions
VA-ECMO	Veno-arterial extracorporeal membrane oxygenation
VSR	Ventricular septal rupture

## References

[1] Damluji A.A., van Diepen S., Katz J.N., Menon V., Tamis-Holland J.E., Bakitas M., Cohen M.G., Balsam L.B., Chikwe J., American Heart Association Council on Clinical Cardiology (2021). Mechanical Complications of Acute Myocardial Infarction: A Scientific Statement From the American Heart Association. Circulation.

[2] Crenshaw B.S., Granger C.B., Birnbaum Y., Pieper K.S., Morris D.C., Kleiman N.S., Vahanian A., Califf R.M., Topol E.J. (2000). Risk factors, angiographic patterns, and outcomes in patients with ventricular septal defect complicating acute myocardial infarction. GUSTO-I (Global Utilization of Streptokinase and TPA for Occluded Coronary Arteries) Trial Investigators. Circulation.

[3] Elbadawi A., Elgendy I.Y., Mahmoud K., Barakat A.F., Mentias A., Mohamed A.H., Ogunbayo G.O., Megaly M., Saad M., Omer M.A. (2019). Temporal Trends and Outcomes of Mechanical Complications in Patients with Acute Myocardial Infarction. JACC Cardiovasc. Interv..

[4] Cubeddu R.J., Lorusso R., Ronco D., Matteucci M., Axline M.S., Moreno P.R. (2024). Ventricular Septal Rupture After Myocardial Infarction: JACC Focus Seminar 3/5. J. Am. Coll. Cardiol..

[5] Morimura H., Tabata M. (2020). Delayed surgery after mechanical circulatory support for ventricular septal rupture with cardiogenic shock. Interact. Cardiovasc. Thorac. Surg..

[6] Arsh H., Pahwani R., Arif Rasool Chaudhry W., Khan R., Khenhrani R.R., Devi S., Malik J. (2023). Delayed Ventricular Septal Rupture Repair After Myocardial Infarction: An Updated Review. Curr. Probl. Cardiol..

[7] Rashid H., Kumar K., Ullah A., Kamin M., Shafique H.M., Elahi A., Najam A., Zaidi S.M.J., Asad M., Mahmoodi A. (2023). Delayed Ventricular Septal Rupture Repair on Patient Outcomes After Myocardial Infarction: A Systematic Review. Curr. Probl. Cardiol..

[8] Luo W., Wen L., Zhang J., Zhao J., Wang Z., Luo X., Pi S., Chen Y., Zhang J., Li T. (2024). The short-term outcomes and risk factors of post-myocardial infarction ventricular septal rupture: A multi-center retrospective Study. J. Cardiothorac. Surg..

[9] Wang S., Zhang J., Xiao Q.F., Liu K., Xu Y., Chen X.P., Wei X., Peng Y. (2023). Risk factors for immediate and delayed cardiogenic shock in patients with ventricular septal rupture after myocardial infarction. Front. Cardiovasc. Med..

[10] Byrne R.A., Rossello X., Coughlan J.J., Barbato E., Berry C., Chieffo A., Claeys M.J., Dan G.A., Dweck M.R., Galbraith M. (2023). 2023 ESC Guidelines for the management of acute coronary syndromes. Eur. Heart J..

[11] Chinese Society of Cardiology of Chinese Medical Association, Editorial Board of Chinese Journal of Cardiology (2019). 2019 Chinese Society of Cardiology (CSC) guidelines for the diagnosis and management of patients with ST-segment elevation myocardial infarction. Zhonghua Xin Xue Guan Bing Za Zhi.

[12] Ronco D., Matteucci M., Ravaux J.M., Marra S., Torchio F., Corazzari C., Massimi G., Beghi C., Maessen J., Lorusso R. (2021). Mechanical Circulatory Support as a Bridge to Definitive Treatment in Post-Infarction Ventricular Septal Rupture. JACC Cardiovasc. Interv..

[13] Matteucci M., Ronco D., Corazzari C., Fina D., Jiritano F., Meani P., Kowalewski M., Beghi C., Lorusso R. (2021). Surgical Repair of Postinfarction Ventricular Septal Rupture: Systematic Review and Meta-Analysis. Ann. Thorac. Surg..

[14] Arnaoutakis G.J., Zhao Y., George T.J., Sciortino C.M., McCarthy P.M., Conte J.V. (2012). Surgical repair of ventricular septal defect after myocardial infarction: Outcomes from the Society of Thoracic Surgeons National Database. Ann. Thorac. Surg..

[15] Hua K., Peng Z., Yang X. (2021). Long-Term Survival and Risk Factors for Post-Infarction Ventricular Septal Rupture. Heart Lung Circ..

[16] Wang L., Xiao L.L., Liu C., Zhang Y.Z., Zhao X.Y., Li L., Wang X.F., Dong J.Z. (2021). Clinical Characteristics and Contemporary Prognosis of Ventricular Septal Rupture Complicating Acute Myocardial Infarction: A Single-Center Experience. Front. Cardiovasc. Med..

[17] Sinha S.S., Morrow D.A., Kapur N.K., Kataria R., Roswell R.O. (2025). 2025 Concise Clinical Guidance: An ACC Expert Consensus Statement on the Evaluation and Management of Cardiogenic Shock: A Report of the American College of Cardiology Solution Set Oversight Committee. J. Am. Coll. Cardiol..

[18] Ronco D., Matteucci M., Kowalewski M., De Bonis M., Formica F., Jiritano F., Fina D., Folliguet T., Bonaros N., Russo C.F. (2021). Surgical Treatment of Postinfarction Ventricular Septal Rupture. JAMA Netw. Open.

[19] Matteucci M., Fina D., Jiritano F., Meani P., Raffa G.M., Kowalewski M., Aldobayyan I., Turkistani M., Beghi C., Lorusso R. (2020). The use of extracorporeal membrane oxygenation in the setting of postinfarction mechanical complications: Outcome analysis of the Extracorporeal Life Support Organization Registry. Interact. Cardiovasc. Thorac. Surg..

[20] Shibasaki I., Otani N., Saito S., Ogawa H., Masawa T., Tsuchiya G., Takei Y., Tezuka M., Kanazawa Y., Kanno Y. (2023). Overview of mechanical circulatory support for the management of post-myocardial infarction ventricular septal rupture. J. Cardiol..

[21] Thiele H., Zeymer U., Neumann F.J., Ferenc M., Olbrich H.G., Hausleiter J., Richardt G., Hennersdorf M., Empen K., Fuernau G. (2012). Intraaortic balloon support for myocardial infarction with cardiogenic shock. N. Engl. J. Med..

[22] Fuernau G., Ledwoch J., Desch S., Eitel I., Thelemann N., Jung C., de Waha-Thiele S., Poss J., Feistritzer H.J., Freund A. (2021). Impact of timing of intraaortic balloon counterpulsation on mortality in cardiogenic shock—A subanalysis of the IABP-SHOCK II trial. Eur. Heart J. Acute Cardiovasc. Care.

[23] Yuan L., Nie S.P. (2016). Efficacy of Intra-aortic Balloon Pump before versus after Primary Percutaneous Coronary Intervention in Patients with Cardiogenic Shock from ST-elevation Myocardial Infarction. Chin. Med. J..

[24] Russo J.J., Del Sorbo L. (2023). VA-ECMO When All Seems Lost: Defining the Right Person, Place, and Time. J. Am. Coll. Cardiol..

[25] Kapur N.K., Hall S. (2023). Time Is “Not” on Your Side When Managing Cardiogenic Shock, a Loaded Ventricle, and VA-ECMO. JACC Heart Fail..

[26] Rao S.V., O’Donoghue M.L., Ruel M., Rab T., Tamis-Holland J.E., Alexander J.H., Baber U., Baker H., Cohen M.G., Cruz-Ruiz M. (2025). 2025 ACC/AHA/ACEP/NAEMSP/SCAI Guideline for the Management of Patients with Acute Coronary Syndromes: A Report of the American College of Cardiology/American Heart Association Joint Committee on Clinical Practice Guidelines. Circulation.

[27] Pahuja M., Schrage B., Westermann D., Basir M.B., Garan A.R., Burkhoff D. (2019). Hemodynamic Effects of Mechanical Circulatory Support Devices in Ventricular Septal Defect. Circ. Heart Fail..

[28] Jiritano F., Lo Coco V., Matteucci M., Fina D., Willers A., Lorusso R. (2020). Temporary mechanical circulatory support in acute heart failure. Card. Fail. Rev..

[29] Giblett J.P., Matetic A., Jenkins D., Ng C.Y., Venuraju S., MacCarthy T., Vibhishanan J., O’Neill J.P., Kirmani B.H., Pullan D.M. (2022). Post-infarction ventricular septal defect: Percutaneous or surgical management in the UK national registry. Eur. Heart J..

[30] Malhotra A., Patel K., Sharma P., Wadhawa V., Madan T., Khandeparkar J., Shah K., Patel S. (2017). Techniques, Timing & Prognosis of Post Infarct Ventricular Septal Repair: A Re-look at Old Dogmas. Braz. J. Cardiovasc. Surg..

[31] Hu X.Y., Qiu H., Qiao S.B., Kang L.M., Song L., Zhang J., Tan X.Y., Wu Y., Yang Y.J., Gao R.L. (2013). Clinical analysis and risk stratification of ventricular septal rupture following acute myocardial infarction. Chin. Med. J..

[32] Ciuca-Pana M.A., Boulmpou A., Ileri C., Manzi G., Golino M., Ostojic M., Galimzhanov A., Busnatu S., Mega S., Perone F. (2025). Chronic Heart Failure and Coronary Artery Disease: Pharmacological Treatment and Cardiac Rehabilitation. Medicina.

